# Impact of baseline ^18^F-flotufolastat PET bone tumor volume for prognosticating severe hematologic toxicity in patients with metastatic castration-resistant prostate Cancer receiving ^177^Lu-PSMA-targeted radioligand therapy

**DOI:** 10.1007/s00259-025-07200-7

**Published:** 2025-05-19

**Authors:** Amir Karimzadeh, Kimberley Hansen, Stefan Hein, Bernhard Haller, Matthias M. Heck, Robert Tauber, Calogero D`Alessandria, Matthias Eiber, Isabel Rauscher

**Affiliations:** 1 Department of Nuclear Medicine, School of Medicine and Health, TUM University Hospital, Munich, Germany; 2 Department of Diagnostic and Interventional Radiology and Nuclear Medicine, University Medical Center Hamburg-Eppendorf, Martinistr. 52, 20246 Hamburg, Germany; 3 School of Medicine and Health, Institute of AI and Informatics in Medicine, Technical University of Munich, TUM University Hospital, Munich, Germany; 4 Department of Urology, School of Medicine and Health, TUM University Hospital, Munich, Germany; 5 Bavarian Cancer Research Center, Munich, Germany

**Keywords:** [^177^Lu]Lu-PSMA-I&T; mCRPC, Hematologic toxicity, Bone tumor metrics, PSMA radioligand therapy

## Abstract

**Purpose:**

This retrospective analysis evaluated the prognostic value of baseline ^18^F-flotufolastat-PET bone tumor metrics for severe hematologic toxicity in metastatic castration-resistant prostate cancer (mCRPC) patients treated with [^177^Lu]Lu-PSMA-I&T.

**Methods:**

Data from 182 mCRPC patients with baseline ^18^F-flotufolastat-PET scans and complete hematologic profiles were analyzed. Bone lesions were semiautomatically delineated, and clinical parameters (e.g., pretreatments, lab results) were assessed. Hematologic adverse events (AEs) were defined per Common Terminology Criteria for Adverse Events version 5.0, with grades 3–4 considered severe. Cox regression was used to identify prognostic factors for AEs.

**Results:**

Baseline bone tumor volume prognosticated leukocytopenia (HR 1.03 per 100 ml, *p* = 0.036), while the number of bone lesions was prognostic for anemia (HR 1.04 per 10 lesions, *p* < 0.001) and severe anemia (HR per 10 lesions 1.05, *p* = 0.009). Higher baseline hemoglobin correlated with reduced leukocytopenia (HR 0.74, *p* = 0.002), thrombocytopenia (HR 0.80, *p* = 0.033), and severe anemia (HR 0.52, *p* < 0.001). Baseline kidney dysfunction was linked to anemia (HR 2.46, *p* = 0.002) and severe anemia (HR 3.81, *p* = 0.023). Prior [^223^Ra]Radiumdichloride treatment prognosticated severe thrombocytopenia (HR 6.43, *p* = 0.021).

**Conclusion:**

Baseline ^18^F-flotufolastat-PET metrics and pretherapeutic clinical parameters are key prognostic factors for severe hematologic toxicity in mCRPC patients treated with [^177^Lu]Lu-PSMA-I&T.

**Supplementary Information:**

The online version contains supplementary material available at 10.1007/s00259-025-07200-7.

## Clinical trial number

Not applicable.

**Supplementary Information:**

The online version contains supplementary material available at 10.1007/s00259-025-07200-7.

## Introduction

Lutetium-177-(^177^Lu)- prostate-specific membrane antigen (PSMA)-targeted radioligand therapy (RLT) has become an established treatment option for patients with metastatic castration-resistant prostate cancer (mCRPC). Initially supported by data from compassionate use programs, subsequent phase II and III trials have confirmed the therapeutic efficacy of ^177^Lu-PSMA-RLT, leading to regulatory approvals worldwide [1–6]. While the toxicity profile of ^177^Lu- PSMA-RLT is generally considered low, bone marrow suppression is among the most common treatment-emergent adverse events (AEs), not infrequently leading to treatment interruptions or discontinuation of therapy.

The VISION trial reported bone marrow suppression in 47% of patients in the [^177^Lu]Lu-PSMA-617 arm, with 23% experiencing high-grade suppression (Grade 3 or higher) according to the Common Terminology Criteria for Adverse Events version 5.0 (CTCAE v 5.0) [5, 7]. The TheraP trial similarly reported severe hematologic AEs, with anemia in 8% of patients, thrombocytopenia in 11%, and leukocytopenia in 1% [4].

Two key effects are hypothesized to explain bone marrow suppression during ^177^Lu- PSMA-RLT. First, bone marrow suppression may result from direct radiation absorbed by the marrow combined with cross-dose exposure from radioligand in surrounding tissues, an effect especially pronounced in patients with diffuse bone metastases [8]. Second, an increasingly extensive osseous tumor burden, often present in late stage mCRPC, may displace bone marrow, impair its function, and further reduce hematopoiesis [9]. While recent analyses demonstrate a link between hematologic toxicity and the extent of PSMA-expressing bone tumor volume (TV) in patients with mCRPC [10], the specific prognostic value of baseline PSMA-targeted positron emission tomography (PET) parameters for prognosticating severe hematologic AEs that are critical for decision making during ^177^Lu- PSMA-RLT remains uncertain. To the best of our knowledge, this relationship has only been explored in preliminary analyses, yielding limited and inconsistent findings, underscoring the need for further investigation [11, 12].

Therefore, this retrospective analysis aimed to assess the prognostic impact of PSMA-ligand PET with ^18^F-flotufolastat [13] on the occurrence of severe hematologic AEs in a large cohort of patients with mCRPC undergoing ^177^Lu- PSMA-RLT.

## Materials and methods

### Patients and 177Lu-PSMA-RLT

In this retrospective, single-center analysis, data from patients with mCRPC treated with [^177^Lu]Lu-PSMA-I&T at our clinic between November 2017 and September 2021 were reviewed. Of note, data on treatment response and survival outcomes of this patient population has been previously published (DOI 10.1007/s00259-024-07003-2). Patients received RLT with a standard dose of approximately 7.4 GBq [^177^Lu]Lu-PSMA-I&T in a median interval of 6 weeks (interquartile range (IQR) 6–7 weeks) with minor dose adjustments based on factors such as lab results and tumor burden. Baseline ^18^F-flotufolastat PET scans were available for all patients, and eligibility required PSMA-ligand uptake in tumor lesions at least as high as liver background levels. [^177^Lu]Lu-PSMA-I&T was produced according to good manufacturing practices and the German Medicinal Products Act (AMG § 13 2b). Written informed consent was obtained from all patients, and treatments were conducted in line with article 37 of the Declaration of Helsinki regarding unproven interventions in clinical practice. This analysis was approved by the institutional ethics committee (reference number 115/18S).

### ^18^F-Flotufolastat PET imaging procedure

^18^F-Flotufolastat radiolabeling was performed as previously described [14], in compliance with the German Medicinal Products Act, AMG § 13 2b. Written informed consent was obtained from all patients. ^18^F-Flotufolastat was administered via intravenous bolus with a mean activity of 309 ± 65 MBq and PET was performed at a median time of 70 min (IQR 65–79 min) post-injection. Patients received an oral contrast medium (300 mg Telebrix; Guerbet) and 10 mg of furosemide. PET/computed tomography (CT) was conducted using either a Biograph mCT Flow scanner or a Biograph Vision scanner (Siemens Medical Solutions). PET/CT scans were acquired in 3D mode, with acquisition rates of 0.8 mm/s for the mCT Flow scanner and 1.1 mm/s for the Vision scanner. PET images were reconstructed using ordered-subset expectation maximization (TrueX, 3 iterations, 21 subsets for the mCT Flow scanner and 4 iterations, 5 subsets for the Vision scanner) and post-reconstruction Gaussian smoothing (2 mm and 5 mm full width at half maximum for the mCT Flow and Vision scanner, respectively). A diagnostic CT scan was initially performed in the portal venous phase, 80 s after intravenous injection of an iodinated contrast agent (Imeron 300; Bracco Imaging), followed by the ^18^F-flotufolastat PET scan.

### Baseline^18^F-flotufolastat parameters

The imaging parameters analyzed included the number of bone lesions, TV in bone, intensity-weighted total lesion volume (ITLV) in bone (ITLV = ∑(lesion index × lesion uptake volume)), and the highest maximum standardized uptake value (SUV_max_) in bone lesions [15]. These parameters were evaluated through semiautomatic delineation with aPROMISE (version 1.1) developed by EXINI Diagnostics AB [15, 16]. When necessary, any missed pathological foci were manually added, and PSMA-avid foci due to physiological tracer uptake were excluded. Lesions were then reviewed by an experienced PSMA-ligand PET reader. aPROMISE automates the PROMISE (Prostate Cancer Molecular Imaging Standardized Evaluation) framework, a standardized system for assessing PSMA-ligand PET/CT [17]. To achieve this, aPROMISE employs a three-step segmentation process that integrates anatomical and functional imaging [15, 16]. First, deep learning-based organ segmentation is applied to the CT component to delineate anatomical structures. This step provides a reference framework to differentiate physiological PSMA-ligand uptake from pathological lesions, reducing false positives in high-uptake organs. Next, blob detection algorithms identify PSMA-positive regions within the PET image, enabling the detection of small or atypically located lesions, including those with uptake below conventional SUV-based thresholds. Finally, the fast marching method refines lesion boundaries in an adaptive manner, mitigating over-segmentation and improving accuracy in cases of variable background uptake.

### Pretherapeutic clinical parameters and monitoring of hematologic parameters

The following pretherapeutic clinical parameters were assessed: white blood cell count (WBC), hemoglobin (Hb), platelet count (Plt), age, time since initial diagnosis, prior chemotherapy, previous treatment with Abiraterone and/or Enzalutamide, previous treatment with [^223^Ra]Radiumdichloride, prostate-specific antigen (PSA), and creatinine. Patients were classified as having kidney dysfunction at baseline if they had Grade 1 or higher renal impairment according to CTCAE v 5.0 [7].

Hematologic parameters (WBC, Hb, and Plt) were monitored in all patients at the time of the second treatment cycle, at each subsequent cycle up to the sixth cycle, and during interim PSMA-ligand PET/CT imaging for restaging, including after the sixth cycle. Baseline blood work was predominantly assessed on the day of the first treatment cycle. Laboratory measurements were consistently obtained with a median interval of 6 weeks (IQR 5–6 weeks) following each treatment cycle throughout the observation period. According to CTCAE v 5.0 [7], WBC, Hb and Plt were classified based on each laboratory result. Only patients with complete baseline hematologic profile were included in the analysis.

An AE was defined as a deterioration in hematologic parameters (WBC, Hb, or Plt) by at least one CTCAE grade compared to baseline. Severe hematologic AEs were defined as progression to grade 3 or 4 toxicity in WBC, Hb, or Plt. “Any hematologic event” was defined as the occurrence of at least one such event in any of these parameters, encompassing both a deterioration by at least one CTCAE grade and progression to grade 3 or 4 toxicity.

### Statistical analysis

Continuous covariates are presented as median values with IQR, or mean values ± standard deviation. Categorical covariates are reported as frequencies and proportions. Spearman’s rank correlation coefficient was estimated to quantify the strength of association between quantitative variables. Univariate and multivariate Cox regression analyses were used to model time to first occurrence of an AE (deterioration by at least one CTCAE grade) or a severe AE (progression to grade 3 or 4) in the hematologic parameters (WBC, Hb, or Plt), as well as time to first occurrence of “any hematologic event”. The multivariate analysis included the significant parameters identified in the univariate analysis. Patients with no occurrence of an event were censored at the time of their last lab results during the observation period. Hazard ratios (HR) and 95% confidence intervals (CI) are reported. Tests were performed two-sided and a significance level of 5% was used. Kaplan-Meier curves were used to visualize the probability of experiencing the first hematologic AE by any CTCAE grade and the first severe hematologic AE. If reached, median times to event, the number of events in each group, and HRs with 95% CIs, derived from univariate Cox regression analyses, were included in the plots. Analyses were performed using GraphPad Prism version 10.2.2 (341) for Mac.

## RESULTS

A total of 182 mCRPC patients with baseline ^18^F-flotufolastat PET/CT and complete hematologic profiles were included in this retrospective analysis. Baseline PET/CT showed bone metastases in 93.4% (*n* = 170) of patients, with a median of 114 bone lesions (IQR 29–225) and a median bone TV of 385 mL (IQR 67–1113 mL). Prior treatments included chemotherapy in 71% (*n* = 129) and [^223^Ra]Radiumdichloride in 8.2% (*n* = 15). At baseline, the median Hb was 11.9 g/dL (IQR 10.4–13.1), median PSA was 72.1 ng/mL (IQR 23.0–219.5) and median creatinine was 0.9 mg/dL (IQR 0.8–1.1). At baseline kidney dysfunction affected 17% (*n* = 31 [Grade 1: *n =* 26; Grade 2: *n* = 5]) of patients. For detailed patient characteristics see Table 1.

**Table 1:** Patient characteristics

Characteristic	*N* = 182	
**Age**,** years**	74 (68–79)	
**Time since initial diagnosis**,** years**	6.5 (4.0–11.4)	
**No. of [** ^**177**^ **Lu]Lu-PSMA-I&T cycles**	4 (2–6)	
**Pretherapeutic blood parameters**		
WBC, 10^3^/µL	6.4 (5.36–7.86)	
Hb, g/dL	12 (10.4–13.1)	
Plt, 10^3^/µL	241 (194–296)	
PSA, ng/mL^a^	72 (23.0–219.5)	
Creatinine, mg/dL^b^	0.9 (0.8–1.1)	
GFR, mL/min^b^	81 (62–91)	
**Prior systemic therapies**,** n (%)**		
Chemotherapy	129 (71)	
Docetaxel	127 (70)	
Cabazitaxel	24 (13)	
New hormonal agents	175 (96)	
Abiraterone	156 (86)	
Enzalutamide	115 (61)	
[^223^Ra]Radiumdichloride	15 (8.2)	
**Site of metastasis**,** n (%)**		
Lymph nodes	121 (67)	
Bone	170 (93.4)	
Visceral, overall	38 (21)	
Liver	13 (7)	
Lung/Pleura	19 (10)	
Adrenal	10 (5.5)	
Brain	1 (0.5)	
**Baseline** ^**18**^ **F-flotufolastat PET parameters**		
Number of bone lesions, n	114 (29–225)	
Bone TV, mL	385 (67–1113)	
Bone ITLV, mL	767 (158–2446)	
Highest SUV_max_ in a bone lesion	51 (32–87)	

Data are reported as median (interquartile range) unless otherwise stated

^a^*N* = 181; ^b^*N* = 180

^177^Lu: Lutiteum-177; ^18^F: Fluorine-18; ^223^Ra: Radium-223; GFR: glomerular filtration rate; Hb: hemoglobin; ITLV: intensity-weighted lesion volume; PET: positron emission tomography; Plt: platelet; PSA: prostate-specific antigen; PSMA: prostate-specific membrane antigen; SUV_max_: maximum standardised uptake value; TV: tumor volume; WBC: white blood cell

Detailed information on the hematopoietic baseline status and the incidence of hematologic treatment-emergent adverse events, stratified by tumor volume, are provided in the supplementary material.

### Prognostic factors for the onset of treatment-emergent hematologic events of any grade

The results of univariate Cox regression analysis for risk of hematologic AEs during ^177^Lu-PSMA-RLT are presented in Table 2. Significant prognostic factors for leukocytopenia, thrombocytopenia and “any hematologic event” included the number of bone lesions (HR 1.02 [1.00–1.05], *p* = 0.036; HR 1.03 [1.01–1.05], *p* = 0.011 and HR 1.03 [1.01–1.04], *p* < 0.001, respectively; [HRs per 10 lesions]), bone TV (HR 1.05 [1.02–1.07], *p* < 0.001; HR 1.03 [1.01–1.05], *p* = 0.006 and HR 1.03 [1.01–1.04], *p* < 0.001, respectively; [HRs per 100 ml]), and bone ITLV (HR 1.02 [1.01–1.02], *p* < 0.001; HR 1.01 [1.00–1.02], *p* = 0.005 and HR 1.01 [1.01–1.02], *p* < 0.001, respectively; [HRs per 100 ml]). For anemia, significant prognostic factors included the number of bone lesions (HR 1.04 [1.02–1.05], *p* < 0.001; [HR per 10 lesions]).

**Table 2:** Univariate Cox regression analysis of pretherapeutic parameters and risk of first hematologic AE of any grade

Parameter	Leukocytopenia		Anemia		Thrombocytopenia		Any hematologic event	
	HR [95%CI]	*p*-value	HR [95%CI]	*p*-value	HR [95%CI]	*p*-value	HR [95%CI]	*p*-value
**Bone tumour metrics in** ^**18**^ **F-flotufolastat PET**								
No. of bone lesions, per 10 lesions	1.02 [1.00–1.05]	**0.036**	1.04 [1.02–1.05]	**< 0.001**	1.03 [1.01–1.05]	**0.011**	1.03 [1.01–1.04]	**< 0.001**
Bone TV, per 100 mL	1.05 [1.02–1.07]	**< 0.001**	1.01 [0.98–1.03]	0.593	1.03 [1.01–1.05]	**0.006**	1.03 [1.01–1.04]	**< 0.001**
Bone ITLV, per 100 mL	1.02 [1.01–1.02]	**< 0.001**	1.00 [0.99–1.01]	0.646	1.01 [1.00–1.02]	**0.005**	1.01 [1.01–1.02]	**< 0.001**
Highest SUV_max_ in bone lesions	0.99 [0.92–1.03]	0.505	1.00 [0.95–1.04]	0.881	1.00 [0.94–1.04]	0.934	1.00 [0.97–1.03]	0.843
**Pretherapeutic clinical parameters**								
Age	0.99 [0.96–1.03]	0.666	1.01 [0.97–1.04]	0.709	0.99 [0.95–1.03]	0.504	1.01 [0.98–1.04]	0.52
Time since initial diagnosis	0.98 [0.93–1.02]	0.354	0.99 [0.95–1.03]	0.562	0.95 [0.89–1.00]	0.068	0.99 [0.95–1.02]	0.385
Chemotherapy	1.16 [0.62–2.30]	0.652	1.32 [0.76–2.41]	0.345	1.35 [0.68–2.91]	0.414	0.95 [0.62–1.50]	0.824
Abiraterone and/or Enzalutamide	0.52 [0.03–2.40]	0.517	0.79 [0.13–2.53]	0.742	1.66 [0.36–29.48]	0.616	2.53 [0.80–15.36]	0.195
[^223^Ra]Radiumdichloride	1.36 [0.33–3.83]	0.614	1.11 [0.34–2.72]	0.841	1.30 [0.31–3.65]	0.667	1.11 [0.43–2.35]	0.813
Hb	0.66 [0.55–0.78]	**< 0.001**	0.91 [0.79–1.06]	0.224	0.74 [0.62–0.88]	**< 0.001**	0.82 [0.73–0.93]	**0.001**
PSA, per 10 ng/mL	1.01 [1.00–1.01]	**0.002**	1.00 [1.00–1.01]	0.121	1.01 [1.00–1.01]	**0.012**	1.01 [1.00–1.01]	**< 0.001**
Kidney dysfunction	1.03 [0.44–2.10]	0.95	2.40 [1.34–4.14]	**0.002**	1.34 [0.60–2.70]	0.443	1.37 [0.81–2.23]	0.219

Statistically significant p-values are given in bold

^223^Ra: Radium-223; AE: adverse event; CI: confidence interval; Hb: hemoglobin; HR: hazard ratio; ITLV: intensity-weighted lesion volume; PET: positron emission tomography; PSA: prostate-specific antigen; PSMA: prostatespecific membrane antigen; SUV_max_: maximum standardised uptake value; TV: tumor volume

Results for multivariate Cox regression analyses are presented in Table 3. For leukocytopenia, bone TV (HR 1.03 [1.00–1.06], *p* = 0.036; [HR per 100 ml]; Fig. 2a) remained an independent prognostic factor in multivariate analysis, while for anemia, the number of bone lesions (HR 1.04 [1.02–1.06], *p <* 0.001; [HR per 10 lesions]; Fig. 2b) was identified as an independent prognostic factor.

**Table 3:** Multivariate Cox regression analysis of pretherapeutic parameters and risk of first hematologic AE of any grade

Parameter	Leukocytopenia		Anemia		Thrombocytopenia		Any hematologic event		
	HR [95%CI]	*p*-value	HR [95%CI]	*p*-value	HR [95%CI]	*p*-value	HR [95%CI]	*p*-value	
**Bone tumour metrics in** ^**18**^ **F-flotufolastat PET**									
No. of bone lesions, per 10 lesions	0.99 [0.96–1.02]	0.637	1.04 [1.02–1.06]	**< 0.001**	1.02 [0.99–1.04]	0.28	1.02 [1.00–1.04]	0.062	
Bone TV, per 100 mL	1.03 [1.00–1.06]	**0.036**			1.01 [0.98–1.04]	0.551	1.01 [0.99–1.03]	0.304	
**Pretherapeutic clinical parameters**									
Hb	0.74 [0.60–0.89]	**0.002**			0.80 [0.65–0.98]	**0.033**	0.91 [0.78–1.04]	0.167	
PSA, per 10 ng/mL	1.00 [1.00–1.01]	0.2			1.00 [1.00–1.01]	0.551	1.00 [1.00–1.01]	0.067	
Kidney dysfunction			2.46 [1.35–4.30]	**0.002**					

Statistically significant values are given in bold. Due to its high correlation with bone TV, the volume-based metric, bone ITLV, was excluded from multivariate analysis, as it provided no additional prognostic benefit (Fig. S2)

AE: adverse event; CI: confidence interval; Hb: hemoglobin; HR: hazard ratio; ITLV: intensity-weighted lesion volume; NA: not applicable; PET: positron emission tomography; PSA: prostate-specific antigen; PSMA: prostatespecific membrane antigen; SUV_max_: maximum standardised uptake value; TV: tumor volume

**Fig. 1:**
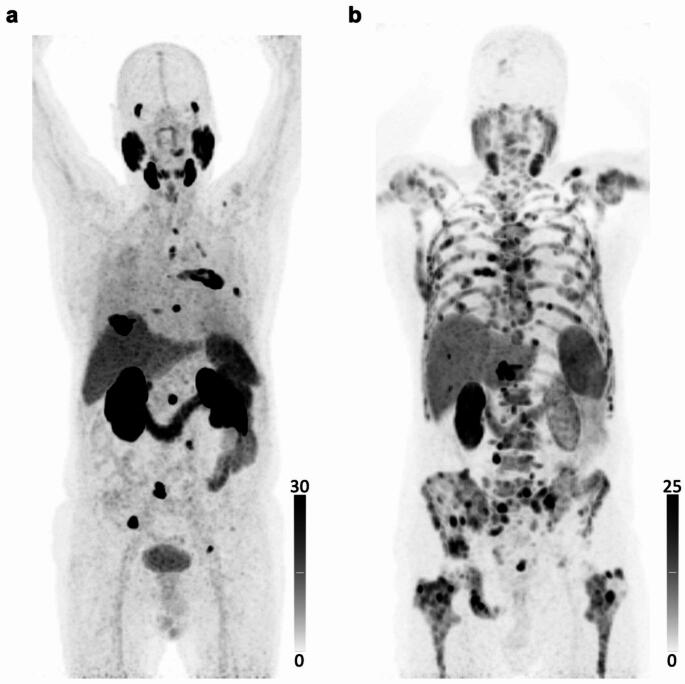
Maximum-intensity projections of [^18^F]-flotufolastat PET in (**a**) a 78-year-old patient with bone metastases, showing 36 bone lesions with a TV of 84 mL, and (**b**) an 81-year-old patient with lymph node and bone metastases with diffuse bone marrow infiltration, showing 268 bone lesions with a TV of 2611 mL. The patient in (**a**) presented with Grade 1 anemia at baseline, no impairment in WBC or Plt, and demonstrated no deterioration in hematopoiesis during treatment. In contrast, the patient in (**b**) presented with Grade 2 thrombocytopenia and Grade 1 anemia at baseline, with no WBC impairment, and subsequently developed CTCAE Grade 3 anemia and thrombocytopenia as well as Grade 1 leukocytopenia by the second treatment cycle, 6 weeks after treatment initiation

**Fig. 2:**
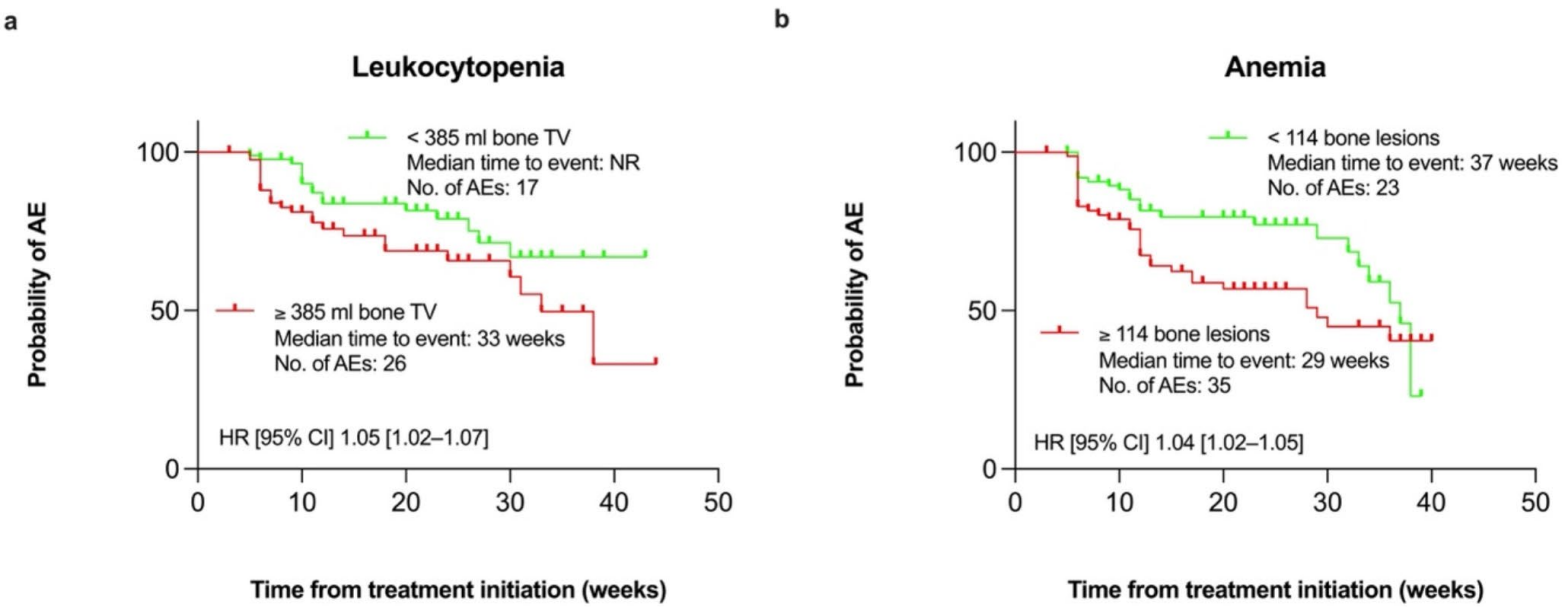
Kaplan-Meier curves for patients with bone metastases (*n* = 170) showing the probability of experiencing the first treatment-emergent hematologic AEs during ^177^Lu-PSMA-RLT for leukocytopenia (**a**) and anemia (**b**). The thresholds used for stratification are based on median values: for leukocytopenia, patients are grouped by bone TV < 385mL (green line) versus ≥ 385 mL: (red line); for anaemia, patients are grouped by having < 114 bone lesions (green line) versus ≥ 114 bone lesions (red line). HR with 95% CI were derived from univariate Cox regression analysis. ^177^Lu: Lutiteum-177; AE: adverse event; CI: confidence interval; HR: hazard ratio; PSMA: prostate-specific membrane antigen; RLT: radioligand therapy; TV: tumor volume

Pretherapeutic clinical parameters associated with leukocytopenia, thrombocytopenia and any hematologic event were Hb (HR 0.66 [0.55–0.78], *p* < 0.001; HR 0.74 [0.62–0.88], *p* < 0.001 and HR 0.82 [0.73–0.93], *p* = 0.001, respectively) and PSA (HR 1.01 [1.00–1.01], *p* = 0.002; HR 1.01 [1.00–1.01], *p* = 0.012 and HR 1.01 [1.00–1.01], *p* < 0.001, respectively). Kidney dysfunction (HR 2.40 [1.34–4.14], *p* = 0.002) was significantly associated with anemia.

In the multivariate analysis, higher Hb levels remained significantly associated with lower rates of leukocytopenia (HR 0.74 [0.60–0.89], *p* = 0.002) and thrombocytopenia (HR 0.80 [0.65–0.98], *p* = 0.033), while kidney dysfunction remained significantly associated with anemia (HR 2.46 [1.35–4.30], *p* = 0.002).

### Prognostic factors of the onset of severe treatment-emergent hematologic events

The results of univariate Cox regression analysis for severe hematologic AEs during ^177^Lu-PSMA-RLT are presented in Table 4. For severe anemia, significant prognostic factors included the number of bone lesions (HR 1.07 [1.04–1.11], *p* < 0.001; [HR per 10 lesions]). For severe thrombocytopenia and “any hematologic event”, significant prognostic factors were the number of bone lesions (HR 1.07 [1.03–1.11], *p* < 0.001 and HR 1.07 [1.04–1.10], *p* < 0.001; [HRs per 10 lesions]), bone TV (HR 1.06 [1.02–1.09], *p* < 0.001 and HR 1.04 [1.01–1.06], *p* = 0.005; [HRs per 100 ml]), and bone ITLV (HR 1.02 [1.01–1.03], *p* < 0.001 and HR 1.01 [1.00–1.02], *p* = 0.009; [HRs per 100 ml]).

**Table 4:** Univariate Cox regression analysis of pretherapeutic parameters and risk of severe hematologic AEs

Parameter	Leukocytopenia		Anemia		Thrombocytopenia		Any hematologic event	
	HR [95%CI]	*p*-value	HR [95%CI]	*p*-value	HR [95%CI]	*p*-value	HR [95%CI]	*p*-value
**Bone tumour metrics in** ^**18**^ **F-flotufolastat PET**								
No. of bone lesions, per 10 lesions	1.10 [0.95–1.29]	0.197	1.07 [1.04–1.11]	**< 0.001**	1.07 [1.03–1.11]	**< 0.001**	1.07 [1.04–1.10]	**< 0.001**
Bone TV, per 100 mL	1.05 [0.88–1.17]	0.428	1.03 [0.99–1.06]	0.072	1.06 [1.02–1.09]	**< 0.001**	1.04 [1.01–1.06]	**0.005**
Bone ITLV, per 100 mL	1.02 [0.96–1.06]	0.322	1.01 [0.99–1.02]	0.176	1.02 [1.01–1.03]	**< 0.001**	1.01 [1.00–1.02]	**0.009**
Highest SUV_max_ in bone lesions	1.00 [0.53–1.17]	0.981	0.84 [0.69–0.98]	0.05	1.00 [0.89–1.07]	0.986	0.94 [0.84–1.03]	0.285
**Pretherapeutic clinical parameters**								
Age	0.96 [0.81–1.15]	0.609	0.98 [0.93–1.05]	0.593	0.96 [0.90–1.04]	0.264	0.98 [0.93–1.03]	0.418
Time since initial diagnosis	0.99 [0.72–1.19]	0.933	0.95 [0.84–1.03]	0.279	0.99 [0.89–1.08]	0.796	0.98 [0.91–1.05]	0.566
Chemotherapy	NA	NA	6.43 [1.28–116.90]	0.073	4.86 [0.93–89.31]	0.132	4.60 [1.33–28.89]	**0.041**
Abiraterone and/or Enzalutamide	NA	NA	NA	NA	NA	NA	NA	NA
[^223^Ra]Radiumdichloride	NA	NA	1.22 [0.07–6.27]	0.85	8.65 [1.79–33.36]	**0.003**	3.26 [0.75–10.05]	0.065
Hb	0.48 [0.18–1.07]	0.09	0.46 [0.33–0.63]	**< 0.001**	0.48 [0.33–0.68]	**< 0.001**	0.46 [0.35–0.60]	**< 0.001**
PSA, per 10 ng/mL	0.99 [0.89–1.01]	0.806	1.01 [1.00–1.01]	**0.026**	1.01 [1.00–1.01]	**0.003**	1.01 [1.00–1.01]	**< 0.001**
Kidney dysfunction	NA	NA	4.80 [1.65–14.06]	**0.003**	2.64 [0.69–8.75]	0.122	2.93 [1.16–6.97]	**0.017**

Statistically significant p-values are given in bold

^223^Ra: Radium-223; AE: adverse event; CI: confidence interval; Hb: hemoglobin; HR: hazard ratio; ITLV: intensity-weighted lesion volume; PET: positron emission tomography; PSA: prostate-specific antigen; PSMA: prostatespecific membrane antigen; SUV_max_: maximum standardised uptake value; TV: tumour volume

**Fig. 3:**
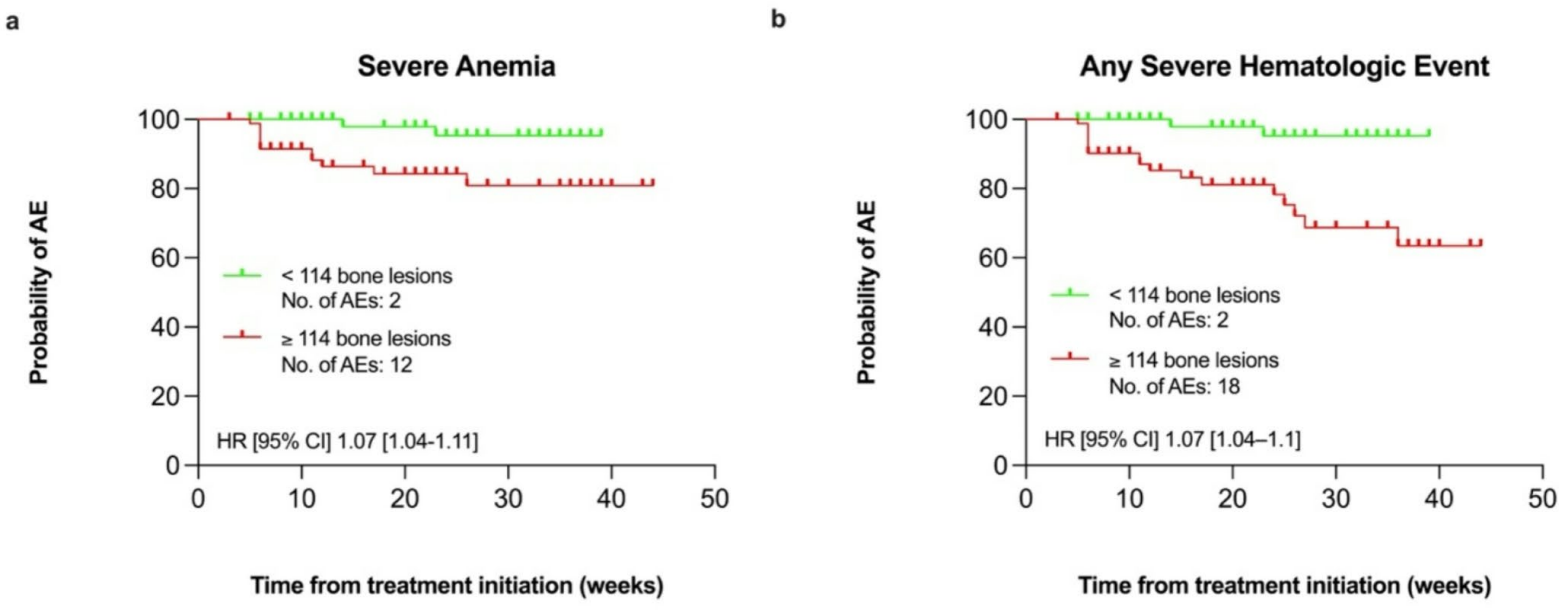
Kaplan-Meier curves for patients with bone metastases (*n* = 170) showing the probability of experiencing the first severe treatment-emergent hematologic AEs during ^177^Lu-PSMA-RLT for severe anemia (**a**) and any severe hematologic event (**b**). Stratification thresholds are based on median values: for severe anemia (**a**) and any severe hematologic event (**b**), patients are grouped by the number of bone lesions < 114 (green line) versus ≥ 114 (red line). HR with 95% CI were derived from univariate Cox regression analysis. ^177^Lu: Lutiteum-177; AE: adverse event; CI: confidence interval; HR: hazard ratio; PSMA: prostate-specific membrane antigen; RLT: radioligand therapy

In the multivariate analysis, the number of bone lesions (HR 1.05 [1.01–1.09], *p* = 0.009; [HR per 10 lesions]; Fig. 3a) was identified as an independent prognostic factor for severe anemia (Table 5). Regarding the risk of “any hematologic event”, significant independent prognostic factor included the number of bone lesions (HR 1.05 [1.01–1.08], *p* = 0.008; [HR per 10 lesions]; Fig. 3b).

**Table 5:** Multivariate Cox regression analysis of pretherapeutic parameters and risk of severe hematologic AEs

Parameter	Anemia		Thrombocytopenia		Any hematologic event	
	HR [95%CI]	*p*-value	HR [95%CI]	*p*-value	HR [95%CI]	*p*-value
**Bone tumour metrics in** ^**18**^ **F-flotufolastat PET**						
No. of bone lesions, per 10 lesions	1.05 [1.01–1.09]	**0.009**	1.05 [0.99–1.1]	0.08	1.05 [1.01–1.08]	**0.008**
Bone TV, per 100 mL			1.04 [0.99–1.08]	0.12	1.02 [0.98–1.05]	0.35
**Pretherapeutic clinical parameters**						
[^223^Ra]Radiumdichloride			6.43 [1.15–30.63]	**0.021**		
Hb	0.52 [0.34–0.75]	**< 0.001**	0.67 [0.43–0.97]	0.051	0.53 [0.38–0.72]	**< 0.001**
PSA, per 10 ng/mL	1.00 [1.00–1.01]	0.672	1.00 [0.99–1.01]	0.979	1.00 [1.00–1.00]	0.747
Kidney dysfunction	3.81 [1.19–12.57]	**0.023**			3.02 [1.05–8.59]	**0.036**

Statistically significant values are given in bold. Due to its high correlation with bone TV, the volume-based metric, bone ITLV, was excluded from multivariate analysis, as it provided no additional prognostic benefit (Fig. S2)

^223^Ra: Radium-223; AE: adverse event; CI: confidence interval; Hb: hemoglobin; HR: hazard ratio; NA: not applicable; PET: positron emission tomography; PSA: prostate-specific antigen; PSMA: prostatespecific membrane antigen; TV: tumor volume

Following pretherapeutic clinical parameters were significantly associated with the risk of severe leukocytopenia: Hb (HR 0.46 [0.33–0.63], *p* < 0.001), PSA (HR 1.01 [1.00–1.01], *p* = 0.026), and kidney dysfunction (HR 4.80 [1.65–14.06], *p* = 0.003). For severe thrombocytopenia, Hb (HR 0.48 [0.33–0.68], *p* < 0.001), PSA (HR 1.01 [1.00–1.01], *p* = 0.003), and [^223^Ra]Radiumdichloride pretreatment (HR 8.65 [1.79–33.36], *p* = 0.003) were identified as significant parameters. Prior chemotherapy (HR 4.6 [1.33–28.89], *p* = 0.041), PSA (HR 1.01 [1.00–1.01], *p* < 0.001), Hb (HR 0.46 [0.35–0.60], *p* < 0.001), and kidney dysfunction (HR 2.93 [1.16–6.97], *p* = 0.017) were significantly associated with “any hematologic event”.

In the multivariate analysis, higher Hb levels remained significantly associated with lower rates of severe anemia (HR 0.52 [0.34–0.75], *p* < 0.001) and any severe hematologic event (HR 0.53 [0.38–0.72], *p* < 0.001), as did kidney dysfunction (HR 3.81 [1.19–12.57], *p* = 0.023 and HR 3.02 [1.05–8.59], *p* = 0.036, respectively). For severe thrombocytopenia, pretreatment with [^223^Ra]Radiumdichloride was the only independent prognostic factor (HR 6.43 [1.15–30.63], *p* = 0.021).

## Discussion

Our findings demonstrated the value of bone tumor metrics on baseline ^18^F-flotufolastat PET as well as clinical parameters for prognosticating hematological AEs during ^177^Lu-PSMA-I&T RLT in mCRPC. Specifically, bone TV and baseline Hb were significantly associated with leukocytopenia: one quarter of patients experienced worsening of WBC, and 1% of patients progressed to Grade 3 leukocytopenia. Furthermore, the number of bone lesions, baseline Hb and kidney dysfunction were significantly associated with anemia: one-third of patients had worsening of Hb and around 8% of patients progressed to Grade 3 anemia. Around one-fifth of patients had worsening of Plt, and 6% of patients progressed to Grade 3 or 4 thrombocytopenia. [^223^Ra]Radiumdichloride pretreatment and baseline Hb were significantly associated with thrombocytopenia.

At baseline, the rates of hematologic impairment in our cohort were comparable to those reported in other studies. Specifically, 74%, 13%, and 2.8% of patients presented with CTCAE Grade 1, 2, or 3 anemia, respectively. This aligns closely with another analysis reporting baseline anemia rates of 67%, 14%, and 3% for Grade 1, 2, and 3, respectively [9]. These data suggest that patients with mCRPC already exhibit compromised hematopoietic reserves at the initiation of PSMA-RLT. This impaired hematopoiesis may be attributed to the advanced stage of the disease itself, which can significantly affect bone marrow function, probably together with prior systematic treatments, which might further weaken hematopoietic capacity.

The prospective phase 3 VISION trial reported leukocytopenia in 13% of patients, thrombocytopenia in 17%, and anemia in 32% of patients with mCRPC treated with [^177^Lu]Lu-PSMA-617 [5]. This is similar to our findings, with thrombocytopenia observed in 22% and anemia in 33% of patients. However, the incidence of leukocytopenia in our analysis was approximately twice as high as that reported in the VISION trial (25% vs. 13%) but remained within the range observed in other analyses, such as 40% [18]. The VISION trial also reported severe AEs (CTCAE Grade ≥ 3), including severe leukocytopenia in 2.5%, severe thrombocytopenia in 7.9%, and severe anaemia in 13% of patients [5]. Consistent with the VISION trial, we observed severe leukocytopenia in 1.1%, severe anemia in 7.7%, and severe thrombocytopenia in 6.1% of patients, also suggesting a comparable rate of severe AEs. Similarly, the TheraP trial reported severe leukocytopenia in 1.1%, severe thrombocytopenia in 11%, and severe anemia in 8% of patients [4], demonstrating comparable toxicities.

Our multivariate Cox regression analysis demonstrated that ^18^F-flotufolastat PET-based bone tumor metrics were significant independent prognostic factors for the development of hematologic AEs. An increase in bone TV per 100 mL was identified as an independent prognostic factor for leukocytopenia (HR 1.03, *p* = 0.036), while an increase in the number of bone lesions per 10 was an independent prognostic factor for anemia (HR 1.04, *p* < 0.001), severe anemia (HR 1.05, *p* = 0.009), and any severe hematologic event (HR 1.05, *p* = 0.008). Our analysis showed that both bone lesion number and bone TV have prognostic value for hematologic toxicity. While lesion number more likely reflects disease dissemination, it does not account for lesion size and total tumor mass. Additionally, bone TV may affect therapy efficacy via the tumor-sink effect, altering radionuclide availability, an aspect not captured by lesion count alone. Given these differences, both metrics likely offer complementary prognostic insights.

Our findings are consistent with a recently published analysis investigating the prognostic impact of bone TV in pre-treatment PSMA-ligand PET/CT. In this study, osseous TV demonstrated prognostic capability for hematologic AEs when stratified by cutoffs for leukocytopenia and thrombocytopenia after one cycle [10]. However, in the multivariate analysis, osseous TV was outperformed by baseline laboratory parameters and did not reach statistical significance. In contrast, our analysis, aside from the influence of baseline Hb, did not assess the impact of other baseline hematologic parameters but instead incorporated additional pretherapeutic clinical factors, such as renal function and prior therapies (e.g., [²²³Ra]Radiumdichloride), to account for potential confounding effects. Furthermore, we evaluated the impact of baseline PSMA-ligand PET-derived bone tumor metrics specifically on the occurrence of severe (Grade 3/4) hematologic AEs, which are particularly relevant for risk stratification in ^177^Lu-PSMA RLT.

In another recently published preliminary analysis, a significant association was reported between bone TV and the risk of severe anemia (OR 1.1, 95% CI 1.05–1.2, *p* < 0.001) as well as severe thrombocytopenia (OR 1.1, 95% CI 1.1–1.2, *p* = 0.001). However, no significant association was observed between baseline bone TV and severe leukocytopenia (OR 1.1, 95% CI 1.0–1.2, *p* = 0.08) [12]. Our results highlight the clinical relevance of baseline osseous tumor burden as a prognostic factor for severe hematologic toxicity in ¹⁷⁷Lu-labeled PSMA RLT. In the VISION trial, adverse events were the second most common reason for treatment discontinuation (*n* = 54/551), following disease progression (*n* = 127/551) [5]. Notably, hematologic treatment-emergent adverse events were the most frequent among all severe adverse events (~ 23.4%) [5]. These findings underscore the need for early risk stratification and support integrating bone tumor metrics into clinical practice. Proactively assessing hematologic toxicity risks could enable timely interventions, such as closer monitoring or supportive care (e.g., blood transfusions, growth factors).

In our analysis, patients stratified by TV who developed severe anemia during therapy (*n* = 14) predominantly had a high TV at baseline (*n* = 11), with the majority also presenting with Grade 2 anemia at baseline (*n* = 8). A similar pattern was observed for severe thrombocytopenia, where most affected patients (*n* = 11) also had a high TV at baseline (*n* = 8), while half of the patients already presented with reduced Plt (Grade 1 and 2). These findings suggest a strong correlation between osseous tumor burden and baseline hematopoietic reserve before treatment initiation and the risk of developing severe hematologic AEs during treatment. Building on this, baseline Hb, demonstrated an independent prognostic impact for most hematologic AEs. Higher Hb at baseline was significantly associated with a lower risk of leukocytopenia (HR 0.74, *p* = 0.002) and thrombocytopenia (HR 0.8, *p* = 0.033), as well as severe anemia (HR 0.52, *p* < 0.001) and “any hematologic event” (HR 0.53, *p* < 0.001). Our findings align with the well-documented protective role of higher baseline Hb levels, which may reflect a greater bone marrow reserve, and their association with improved treatment outcomes during ^177^Lu-PSMA-RLT [18]. Taken together, our findings underscore the importance of evaluating extensive bone lesions and elevated bone TV, particularly in patients with preexisting impaired haematopoiesis, as these factors may further exacerbate bone marrow suppression and increase the risk of severe treatment-emergent hematologic AEs.

Another pretherapeutic clinical parameter with a strong independent association, particularly for the development of anemia, was preexisting kidney dysfunction (HR 2.46, *p* = 0.002 for anemia; and HR 3.81, *p* = 0.023 for severe anemia). One possible explanation for this observation is that kidney dysfunction may increase the risk of hematotoxicity during ^177^Lu-PSMA-RLT [19]. The kidneys are widely recognized as organs at risk during this treatment, primarily due to their role as excretory organs [20]. Impaired renal function may further increase the likelihood of hematologic adverse events. Supporting this hypothesis, a recently published analysis reported dosimetric data for a hemodialysis-dependent mCRPC patient undergoing PSMA-RLT [21]. The red marrow dose in this case was 0.15 Gy/GBq, approximately four times higher than the dose reported in a VISION substudy (0.035 Gy/GBq) [22]. This significant increase is likely attributable to the delayed clearance caused by hemodialysis and may partly explain why patients with impaired renal function are more susceptible to hematotoxic events. In contrast to our findings, a preliminary analysis reported no association between baseline renal impairment and the development of severe hematologic adverse events during ^177^Lu-PSMA-RLT (OR 0.7, *p* = 0.6 for any severe hematologic event; OR 1.3, *p* = 0.8 for severe anemia) [12]. However, due to differing methodological approaches, a direct comparison of these results is not feasible. While our findings highlight a potential role for baseline kidney dysfunction, further analyses are necessary to elucidate its exact impact.

Interestingly, pretreatment with [^223^Ra]Radiumdichloride was strongly associated with an increased risk of severe thrombocytopenia during therapy. [^223^Ra]Radiumdichloride is typically administered to mCRPC patients with predominantly extensive symptomatic osseous tumour burden, which may significantly compromise hematopoietic reserve. Consistent with our findings, published data have reported a correlation between prior [^223^Ra]Radiumdichloride treatment and the occurrence of severe hematologic AE, as well as reduced outcomes in patients undergoing subsequent ^177^Lu-PSMA-RLT [23].

One limitation of this study is its single-center, retrospective design. Lab results were collected with a median interval of 6 weeks, usually at the time of each therapy cycle and the interim PSMA-ligand PET/CT. This approach reflects a deliberate trade-off between the potential limitations of missing interim lab results, which may have influenced the accuracy of our Cox regression analysis, and the inclusion of a larger patient cohort. While comparable toxicity rates with leading prospective studies were observed, the possibility of underestimating hematologic toxicity cannot be excluded. Another limitation within the CTCAE grading system itself is that small changes in borderline laboratory values can lead to disproportionate shifts in CTCAE grades, while more substantial changes in certain cases may remain within the same grade, potentially misrepresenting the clinical impact of these variations. Another limitation of our time-to-first-event approach is that it does not capture recurrent toxicity episodes or changes in tumor burden and hematologic parameters over time, limiting the assessment of cumulative therapy effects and toxicity progression. However, to address this, we plan future analyses to evaluate the dynamic change in TV and its impact on hematologic parameters over multiple treatment cycles. Furthermore, while our analysis deliberately focused on PSMA-ligand PET/CT-driven bone tumor metrics to minimize the confounding influence of whole-body tumor burden, which may more accurately reflect RLT toxicity on the hematopoietic system, a limitation of this approach is that visceral and nodal metastases, which may also contribute to hematologic toxicity through inflammation processes, cytokine-driven marrow suppression, and indirect radiation effects, are not accounted for in our analysis.

## Conclusion

Our analysis suggests bone tumor metrics derived from ^18^F-fotufolastat PET are valuable in prognosticating hematologic AEs during ^177^Lu-PSMA-I&T-RLT. The number of bone lesions demonstrated prognostic impact across most hematologic parameters. Additionally, hematopoietic reserve, as reflected by baseline Hb levels, plays a crucial role in identifying patients at higher risk, underscoring the need for comprehensive assessment of both tumor burden and baseline hematologic status.

## Electronic supplementary material

Below is the link to the electronic supplementary material.

Supplementary Material 1

Supplementary Material 2

Supplementary Material 3

## Data Availability

The datasets supporting the conclusions of this study can be made available on reasonable request.
