# Supplementary material for: Impact of baseline 18F-flotufolastat PET bone tumor volume for prognosticating severe hematologic toxicity in patients with metastatic castration-resistant prostate Cancer receiving 177Lu-PSMA-targeted radioligand therapy
Source: Eur J Nucl Med Mol Imaging. 2025 May 19;52(12):4434–45. doi: 10.1007/s00259-025-07200-7 (PMC12491086; doi:10.1007/s00259-025-07200-7)

**Impact of Baseline ^18^F-Flotufolastat PET Bone Tumor Volume for Prognosticating Severe Hematologic Toxicity in Patients with Metastatic Castration-Resistant Prostate Cancer Receiving ^177^Lu-Labeled PSMA-Targeted Radioligand Therapy**

Amir Karimzadeh^1,2^, Kimberley Hansen^1^, Stefan Hein^1^, Bernhard Haller^3^, Matthias M. Heck^4^, Robert Tauber^4^, Calogero D`Alessandria^1^, Matthias Eiber^1,5^, Isabel Rauscher^1,5^

Original Research

^1^Department of Nuclear Medicine, School of Medicine and Health, TUM University Hospital, Munich, Germany

^2^Department of Diagnostic and Interventional Radiology and Nuclear Medicine, University Medical Center Hamburg-Eppendorf, Hamburg, Germany

^3^Technical University of Munich, School of Medicine and Health, Institute of AI and Informatics in Medicine, TUM University Hospital, Munich, Germany

^4^Department of Urology, School of Medicine and Health, TUM University Hospital, Munich, Germany

^5^Bavarian Cancer Research Center, Munich, Germany

First and corresponding author:

Amir Karimzadeh

Department of Diagnostic and Interventional Radiology

and Nuclear Medicine, University Medical Center Hamburg-Eppendorf

Martinistr. 52, 20246 Hamburg, Germany

Email: [amir.karimzadeh@uke.de](mailto:amir.karimzadeh@uke.de)

**RESULTS**

**Occurrence of hematologic treatment-emergent adverse events stratified by tumor volume**

The groups were stratified by bone TV derived from baseline PSMA-ligand PET/CT, with thresholds based on the median value: <385 mL defined as low TV (*N* = 86) and ≥385 mL as high TV (*N* = 84; Figure 1 and 2). At baseline, in the low TV group, 87.2% (n = 75) of the patients presented with hematopoietic impairment. Among them, WBC abnormalities were observed in 3.5% (*n* = 3) with Grade 1 and 1.2% (*n* = 1) with Grade 2 leukocytopenia. Hb abnormalities were noted in 80.2% (*n* = 69) with Grade 1 and 7.0% (*n* = 6) with Grade 2 anemia. Plt was reduced in 3.5% (*n* = 3) with Grade 1 and 2.3% (*n* = 2) with Grade 2 thrombocytopenia. In the high TV group, 97.6% (n = 82) of the patients presented with hematopoietic impairment. Among them, WBC abnormalities were observed in 4.8% (*n* = 4) with Grade 1 and 1.2% (*n* = 1) with Grade 2 leukocytopenia. Hb abnormalities occurred in 67.9% (*n* = 57) with Grade 1, 21.4% (*n* = 18) with Grade 2, and 6.0% (*n* = 5) with Grade 3 anemia. Plt were reduced in 7.1% (*n* = 6) with Grade 1, 4.8% (*n* = 4) with Grade 2, and 2.4% (*n* = 2) with Grade 3 thrombocytopenia.

Among patients with low TV, cumulative worsening of WBC was observed in 19.8% (*n* = 17), with no CTCAE 3 or 4 cases reported (Figure 2a, Table 3). In the high TV group, cumulative WBC worsening reached 31.0% (*n* = 26). CTCAE Grade 3 or 4 cases remained rare, occurring in 1.2% (*n* = 1), who presented with Grade 2 leukocytopenia at baseline.

For Hb, cumulative worsening in the low TV group was 30.2% (*n* = 26), with CTCAE Grade 3 or 4 cases observed in 3.5% (*n* = 3), of whom two presented with Grade 1 anemia and one with Grade 2 anemia at baseline (Figure 2b). In the high TV group, cumulative Hb worsening reached 38.1% (*n* = 32), with CTCAE Grade 3 or 4 cases rising to 13.1% (*n* = 11), of whom three and eight presented with Grade 1 and Grade 2 anemia at baseline, respectively.

For Plt, cumulative worsening in the low TV group was 16.3% (*n* = 14), with CTCAE Grade 3 or 4 cases observed in 3.5% (*n* = 3), of whom one presented with Grade 1 thrombocytopenia, one with Grade 2 thrombocytopenia, and one with no impairment at baseline (Figure 2c). In the high TV group, cumulative Plt worsening reached 27.4% (*n* = 23), with CTCAE Grade 3 or 4 cases reaching 9.5% (*n* = 8), of whom two presented with Grade 1 thrombocytopenia, two with Grade 2 thrombocytopenia, and four with no impairment at baseline.

**Table S1.** First occurrence of a hematologic AE of any grade, including severe (CTCAE grade 3 or 4) in patients with bone metastases (*N* = 170) stratified by the median of bone TV (low volume <385 ml, high volume ≥385 ml) according to CTCAE v 5.0 for WBC (leukocytopenia), Hb (anemia), and Plt (thrombocytopenia), shown from baseline through all treatment cycles up to the Restaging after the sixth cycle

| **Cycle** | **Parameter** | | **LV**  **Any Grade** | **LV**  **Grade 3/4** | **HV**  **Any Grade** | **HV**  **Grade 3/4** |
| --- | --- | --- | --- | --- | --- | --- |
|  | | WBC | 2.3 (2/86) | 0 | 16.7 (14/84) | 0 |
| 2^nd^ CY | | Hb | 11.6 (10/86) | 1.2 (1/86) | 16.7 (14/84) | 7.1 (6/84) |
|  | | Plt | 5.8 (5/86) | 1.2 (1/86) | 10.7 (9/84) | 3.6 (3/84) |
|  | | WBC | 12.3 (10/81) | 0 | 6.9 (5/72) | 1.4 (1/72) |
| RS/3^rd^ CY | | Hb | 6.2 (5/81) | 1.2 (1/81) | 18.1 (13/72) | 4.2 (3/72) |
|  | | Plt | 8.6 (7/81) | 0 | 9.9 (7/71) | 4.2 (3/71) |
|  | | WBC | 4.3 (2/47) | 0 | 5.0 (2/40) | 0 |
| 4^th^ CY | | Hb | 4.3 (2/47) | 0 | 2.5 (1/40) | 2.5 (1/40) |
|  | | Plt | 0 | 0 | 10.3 (4/39) | 0 |
|  | | WBC | 4.9 (2/41) | 0 | 5.6 (2/36) | 0 |
| RS/5^th^ CY | | Hb | 7.3 (3/41) | 2.4 (1/41) | 2.8 (1/36) | 2.8 (1/36) |
|  | | Plt | 4.9 (2/41) | 2.4 (1/41) | 5.7 (2/35) | 5.7 (2/35) |
|  | | WBC | 4.5 (1/22) | 0 | 4.8 (1/21) | 0 |
| 6^th^ CY | | Hb | 9.1 (2/22) | 0 | 14.3 (3/21) | 0 |
|  | | Plt | 0 | 4.5 (1/22) | 4.8 (1/21) | 0 |
|  | | WBC | 0 | 0 | 9.5 (2/21) | 0 |
| RS* | | Hb | 18.2 (4/22) | 0 | 0 | 0 |
|  | | Plt | 0 | 0 | 0 | 0 |
|  | | WBC | 19.8 (17/86) | 0 | 31.0 (26/84) | 1.2 (1/84) |
| Cum. | | Hb | 30.2 (26/86) | 3.5 (3/86) | 38.1 (32/84) | 13.1 (13/84) |
|  | | Plt | 16.3 (14/86) | 3.5 (3/86) | 27.4 (23/84) | 9.5 (8/84) |

Data are presented as % (n/N). At Restaging/3^rd^ Cycle, 4^th^ Cycle, and Restaging/5^th^ Cycle, one, two and one thrombocyte values, respectively, were missing. *At time of Restaging after 6^th^ Cycle, lab values were missing in 7 patients.

CTCAE: Common Terminology Criteria for Adverse Events; Cum.: Cumulative; CY: Cycle; Hb: hemoglobin; HV: High volume; LV: Low volume; Plt: platelet; RS: Restaging; TV: tumor volume; WBC: white blood cell

**Fig. S1** First occurrence of treatment-emergent hematologic AEs stratified by the median of TV (low volume <385 ml, high volume ≥385 ml) according to CTCAE v 5.0 for WBC (leukocytopenia) (a), Hb (anemia) (b), and Plt (thrombocytopenia) (c). Proportions of patients with first worsening by any CTCAE Grade (solid blue and red bars for low and high TV, respectively) or first occurrence of a CTCAE Grade 3 or 4 AE (hatched blue and red bars for low and high TV, respectively) are shown per cycle. Percentages representing the proportion of patients affected during each cycle are annotated within or directly above the bars. Cumulative proportions of patients with first worsening by any CTCAE Grade are shown as solid lines (blue for low TV, red for high TV), while cumulative proportions of patients with first worsening to CTCAE Grade 3 or 4 are shown as dashed lines (blue for low TV, red for high TV).

**
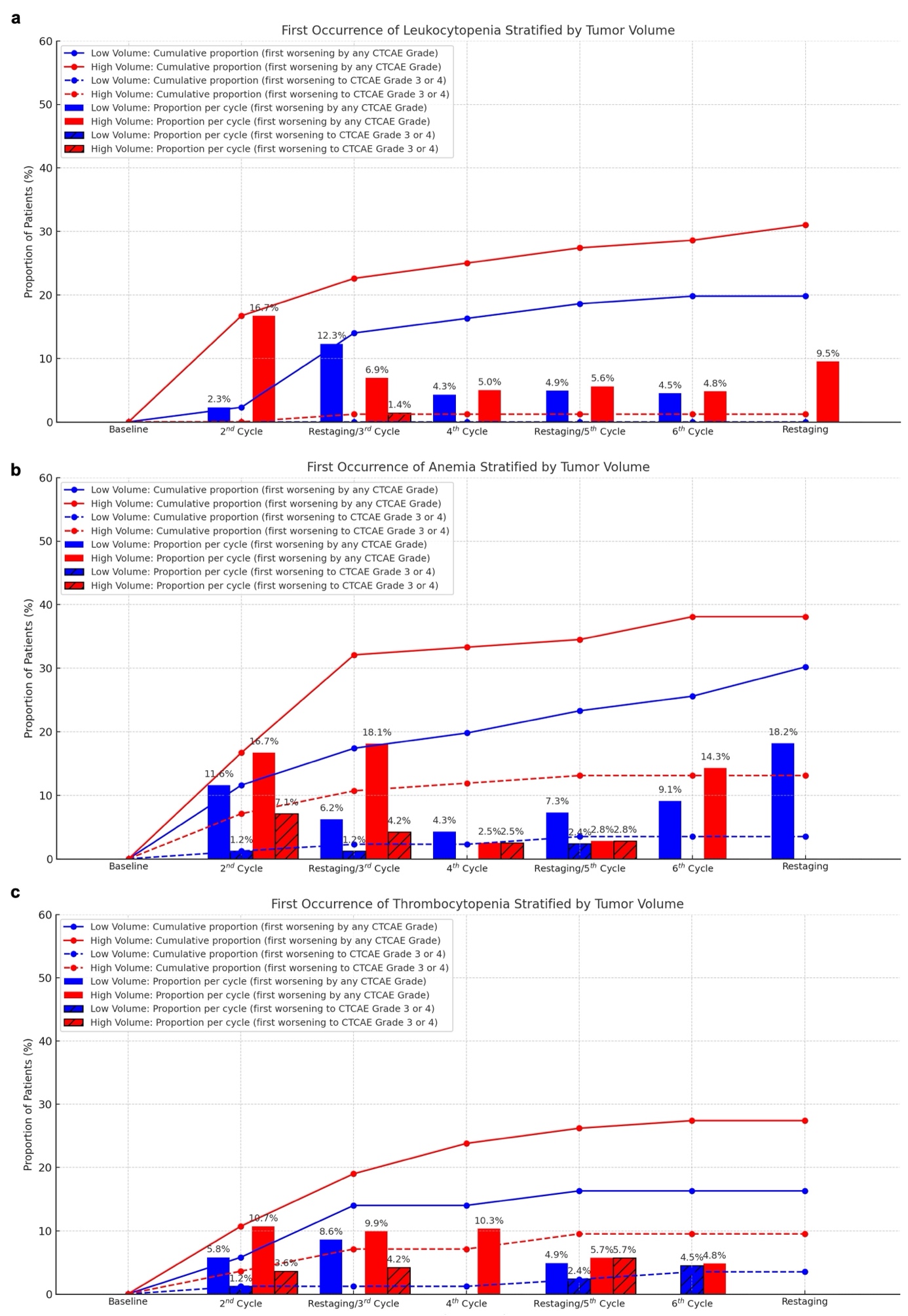
**

At Restaging/3^rd^ Cycle, 4^th^ Cycle, and Restaging/5^th^ Cycle, one, two and two thrombocyte values, respectively, were missing. At time of Restaging after Cycle 6, lab values were missing in 7 patients

AE: adverse event; CTCAE: Common Terminology Criteria for Adverse Events; Hb: hemoglobin; Plt: platelet; PSA: prostate‑specific antigen; PSMA: prostate‑specific membrane antigen; TV: tumor volume; WBC: white blood cell

Fig. S2 Results of Spearman’s rank correlations for TTV and ITLV.


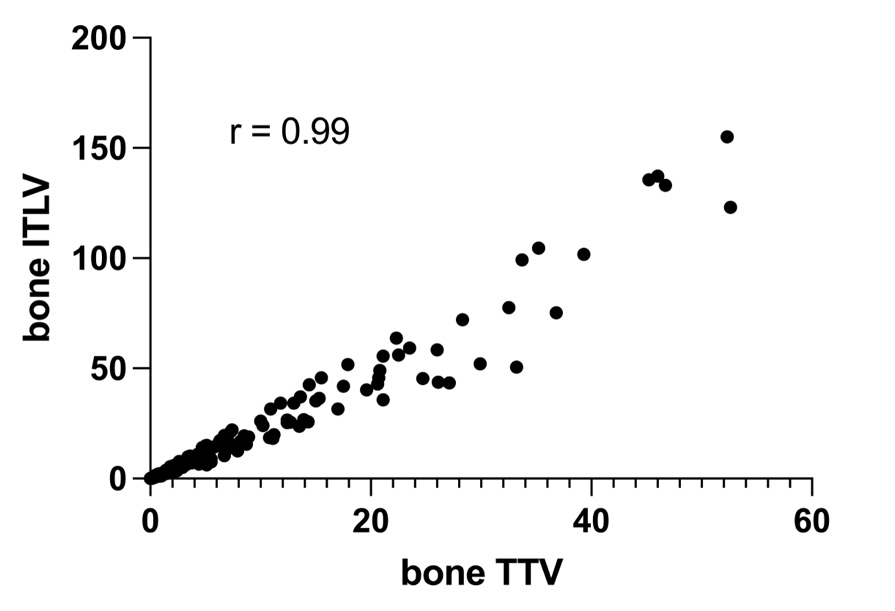

Supplement: Supplementary file 1 — Supplementary Material 1 [file 259_2025_7200_MOESM1_ESM.docx]
