# Supplementary figures and images for: Impact of baseline 18F-flotufolastat PET bone tumor volume for prognosticating severe hematologic toxicity in patients with metastatic castration-resistant prostate Cancer receiving 177Lu-PSMA-targeted radioligand therapy
Source: Eur J Nucl Med Mol Imaging. 2025 May 19;52(12):4434–45. doi: 10.1007/s00259-025-07200-7 (PMC12491086; doi:10.1007/s00259-025-07200-7)

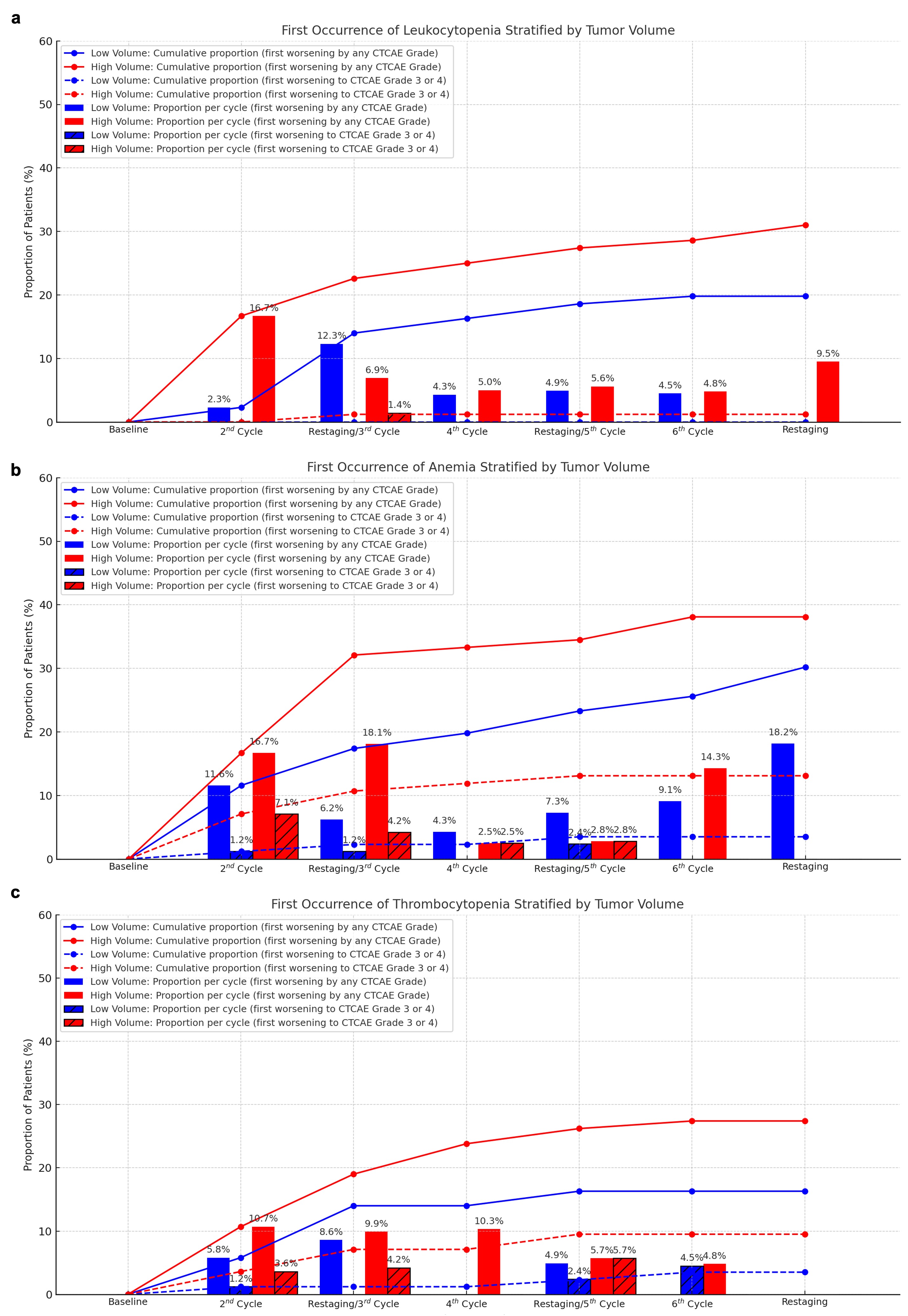

Supplement: Supplementary file 2 — Supplementary Material 2 [file 259_2025_7200_MOESM2_ESM.jpg]

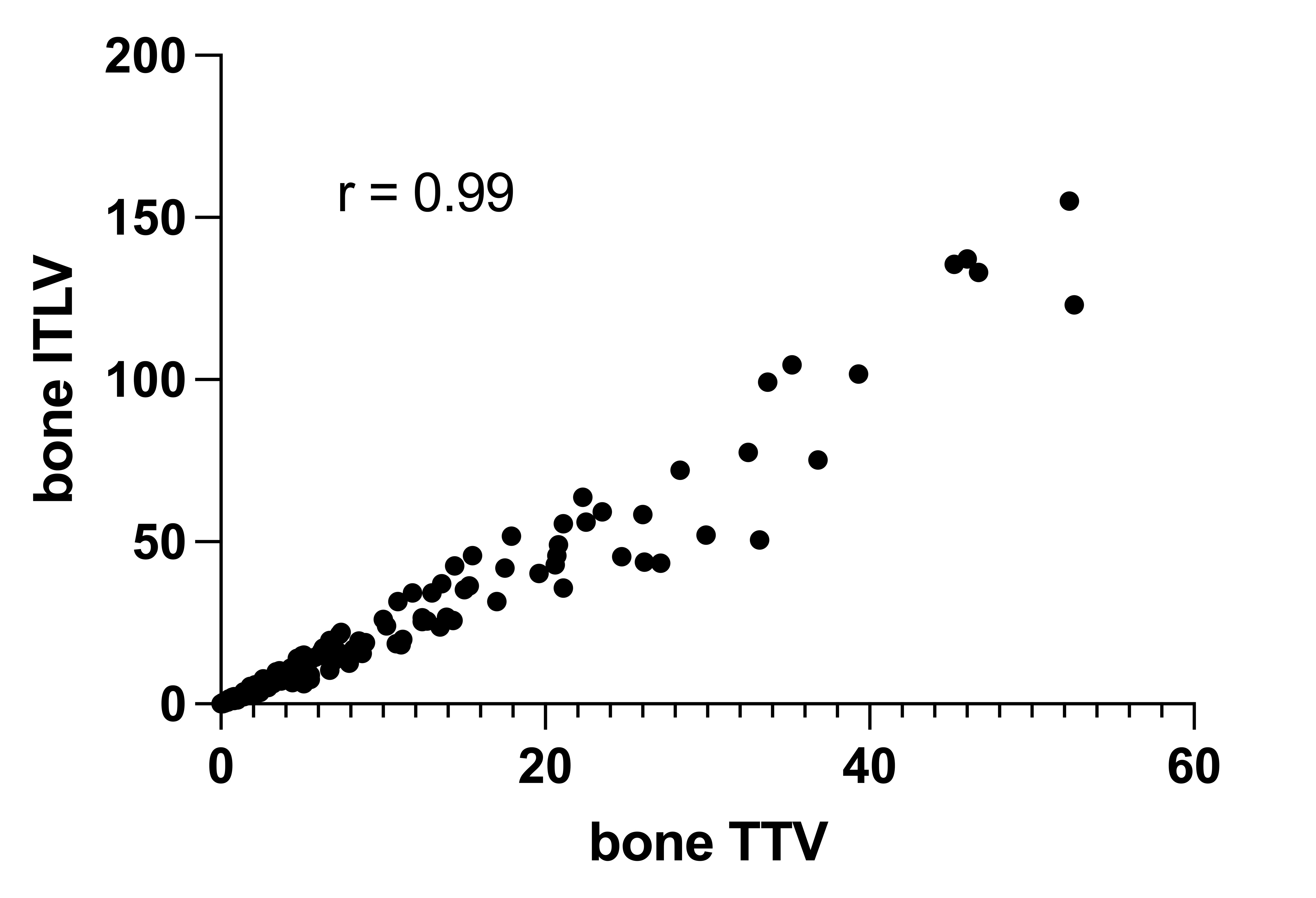

Supplement: Supplementary file 3 — Supplementary Material 3 [file 259_2025_7200_MOESM3_ESM.jpg]
